# Targeting lipid metabolism reprogramming of immunocytes in response to the tumor microenvironment stressor: A potential approach for tumor therapy

**DOI:** 10.3389/fimmu.2022.937406

**Published:** 2022-09-05

**Authors:** Ming Zhang, Tingju Wei, Xiaodan Zhang, Danfeng Guo

**Affiliations:** ^1^ Department of Hepatobiliary and Pancreatic Surgery, the First Affiliated Hospital of Zhengzhou University, Zhengzhou, China; ^2^ Henan Key Laboratory for Digestive Organ Transplantation, Zhengzhou, China; ^3^ Department of Cardiac Surgery, The First Affiliated Hospital of Zhengzhou University, Zhengzhou, China

**Keywords:** tumor microenvironment, lipid metabolism reprogramming, immunocyte, immunotherapy, immunometabolism

## Abstract

The tumor microenvironment (TME) has become a major research focus in recent years. The TME differs from the normal extracellular environment in parameters such as nutrient supply, pH value, oxygen content, and metabolite abundance. Such changes may promote the initiation, growth, invasion, and metastasis of tumor cells, in addition to causing the malfunction of tumor-infiltrating immunocytes. As the neoplasm develops and nutrients become scarce, tumor cells transform their metabolic patterns by reprogramming glucose, lipid, and amino acid metabolism in response to various environmental stressors. Research on carcinoma metabolism reprogramming suggests that like tumor cells, immunocytes also switch their metabolic pathways, named “immunometabolism”, a phenomenon that has drawn increasing attention in the academic community. In this review, we focus on the recent progress in the study of lipid metabolism reprogramming in immunocytes within the TME and highlight the potential target molecules, pathways, and genes implicated. In addition, we discuss hypoxia, one of the vital altered components of the TME that partially contribute to the initiation of abnormal lipid metabolism in immune cells. Finally, we present the current immunotherapies that orchestrate a potent antitumor immune response by mediating the lipid metabolism of immunocytes, highlight the lipid metabolism reprogramming capacity of various immunocytes in the TME, and propose promising new strategies for use in cancer therapy.

## 1 Introduction

Worldwide, an estimated 19.3 million new cancer cases (18.1 million excluding non-melanoma skin cancer) and almost 10.0 million cancer deaths (9.9 million excluding non-melanoma skin cancer) occurred in 2020. The global cancer burden is expected to be 28.4 million cases in 2040, a 47% rise from 2020, with a larger increase in transitioning (64% to 95%) versus transitioned (32% to 56%) countries due to demographic changes, although this may be further exacerbated by increasing risk factors associated with globalization and a growing economy (1). Hence, finding a valid and highly effective therapeutic method for cancer is the primary task for the contemporary medical community.

The tumor microenvironment (TME) has gained recent attention in the field of cancer research, as a complex localized tissue state that comprises various cellular and non-cellular components and soluble molecules (2). Although various diverging neoplasms have been described, the TME is generally characterized by hypoxia, low nutrient levels, and a low pH (3); such changes have been shown to play significant roles in carcinogenesis and tumor progression. Mounting evidence has shown that the TME is correlated with tumor initiation, progression, invasion, metastasis, tumor recurrence, and immune evasion (4). The uncontrolled proliferation of tumor cells depletes blood nutrient and oxygen stores. Such resources are required by the surrounding cells for their normal activity. Additionally, tumor cells also secrete specific effector mediators that construct suitable conditions for their survival.

Immunocytes are pivotal regulators of tumor activity; thus, their normal function directly affects cancer prognosis. The maintenance of normal metabolism is of utmost importance to immunocytes, as their activation, differentiation, and function are dependent on a constant energy supply and metabolic transformation (5). However, due to changes in the availability of fuel and other resources within the TME, immunocytes undergo metabolic pattern alterations that have a profound influence on their immune function. Mounting evidence suggests that immunocytes within the TME exist in an altered metabolic state to survive in such a tough environment (6–8).

Lipids play critical roles in cell function. In addition to being used as an alternative fuel source and resolving energy shortages for cells residing in the TME, lipids also participate in the synthesis of biological membranes, provide substrates for biomass production, and activate complex signaling pathways related to the normal cellular activity (9). Therefore, it is inevitable that cellular function becomes impaired due to aberrant lipid metabolisms, such as the change in cytoplasmic lipid content, fatty acid (FA) oxidation (FAO) and FA synthesis (FAS) levels, and patterns of cholesterol, phospholipid, and lipid droplet (LD) metabolism. The altered lipid metabolism of immunocytes within the TME has raised concerns among the scientific community. Furthermore, a recent study has reported that lipid metabolic alterations in immune cells were commonly associated with the TME and immune dysfunction (6).

The purpose of this review is to provide an overview of recent research progress in the study of lipid metabolism reprogramming of immunocytes within the TME. Specifically, we discuss topics such as the potential markers that may predict the prognosis of patients with cancers and the influence of hypoxia, an oncogenic factor, contributing to the phenomenon of lipid metabolism transition in immunocytes within the TME. The field of cancer immunotherapy has experienced a period of rapid development, with encouraging clinical results and prolonged patient survival, compared with conventional treatment approaches (10). Encouragingly, some forms of immunotherapy have successfully enhanced the antitumor potency of immune cells by regulating their lipid metabolism. These findings support our viewpoint that modulating lipid metabolism in immunocytes is crucial for tumor eradication. Herein, we provide prospective therapeutic strategies by aiming at changes in immunocyte lipid metabolism, which occur as a result of the TME.

## 2 Lipid metabolism reprogramming of various immunocytes in the tumor microenvironment and the associated targets and pathways

The TME is a flexible tissue state comprising a heterogeneous cell population and non-cellular components. TME-associated cell types include precancerous and cancerous cells; more specifically, stromal cells such as epithelial cells, fibroblasts, endothelial cells, and hematopoietically derived immune cells (11). The TME is enriched for the following types of immunocytes: macrophages, T lymphocytes, dendritic cells (DCs), natural killer (NK) cells, myeloid-derived suppressor cells (MDSCs), and neutrophils, among others. Due to the harsh conditions within the TME, these immunocytes are forced to transform their normal metabolic states (regardless of whether they reside within or are recruited to the tumor tissue) to adapt and survive, a process called metabolic reprogramming. Metabolic reprogramming is known to occur in cancer cells and has been suggested as a major sign of cancer progression. In contrast, the metabolic reprogramming of immune cells in the TME has only been recently observed. In the past decade, immunometabolism has progressively become a vibrant area of immunology because of its importance in immunotherapy (12). Nonetheless, we are still some distance from understanding the underlying mechanisms of metabolic changes affecting immunocytes residing in the TME.

**Figure 1:**
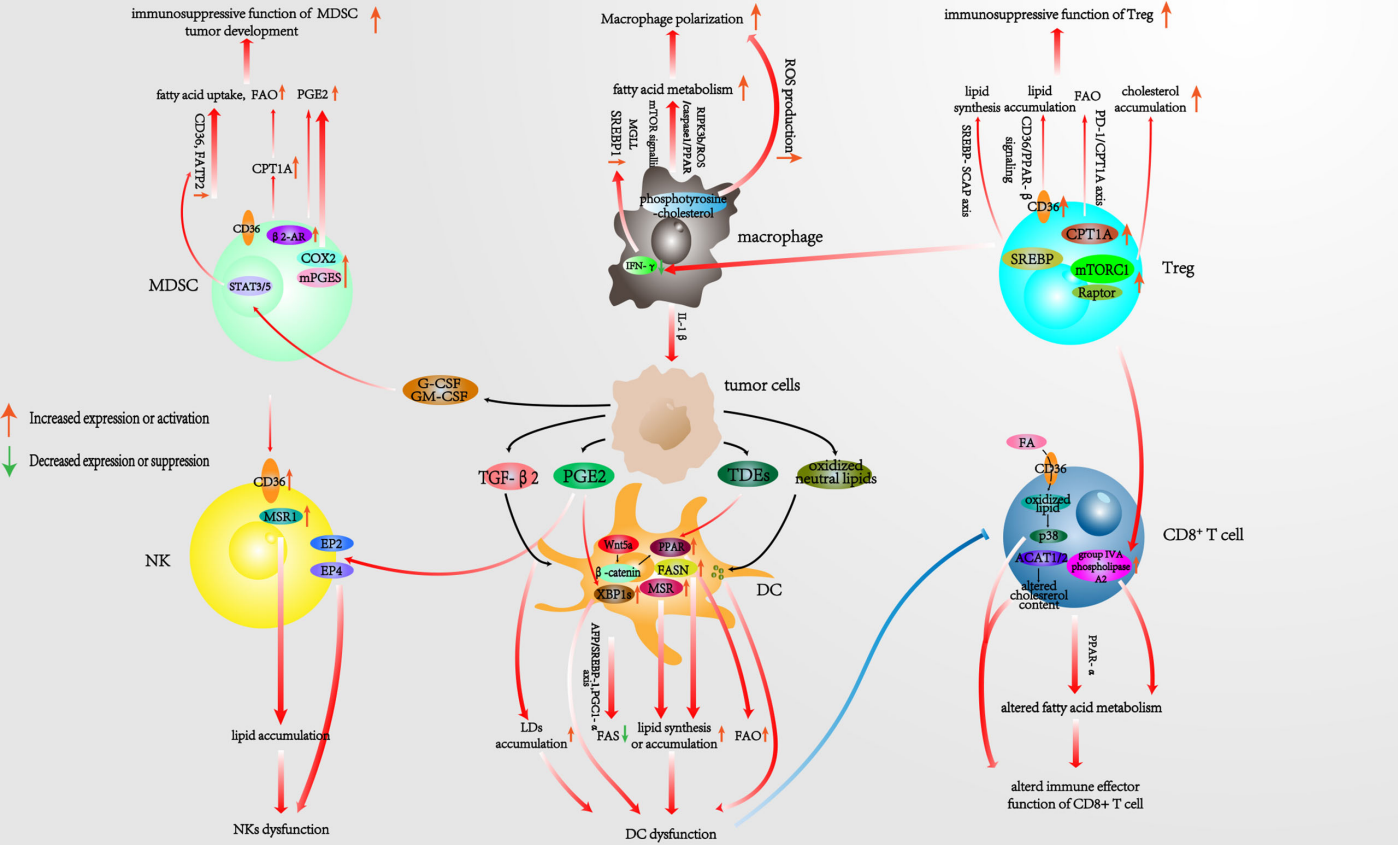
Pathways associated with reprogrammed lipid metabolism of infiltrating immunocytes in the TME. TME, tumor microenvironment.

In general, lipid metabolism involves three major steps: 1) FAS and FAO, 2) steroid metabolism, and 3) compound lipid metabolism. Ever-increasing evidence has shown that the lipid metabolism of tumor-infiltrating immune cells is associated with an immunosuppressive TME and tumor progression (13). We therefore dedicate the following sections of the review to describing the latest research on the signaling axes, proteins, and genes that are associated with lipid metabolism alterations in each type of tumor-infiltrating immunocyte within the TME. Furthermore, we also summarize potential therapeutic targets that are worthy to be considered in this context (**Figure 1**).

### 2.1 Macrophages in the tumor microenvironment

Macrophages carry out multiple critical innate immunity functions. They are essential in immune defense, the inflammatory response, tissue remodeling, and homeostasis. Each of these processes is orchestrated by different macrophage subsets, which display remarkable heterogeneity (14). Macrophages have considerable plasticity and can adopt different activation states in response to changes in their tissue microenvironment (15, 16). They differentiate into distinct phenotypes following stimulation with various factors. These phenotypes exhibit different characteristics and biological functions, thus exerting different regulatory functions for physiological and pathological activities in the body, a phenomenon known as the “polarizing effect” (16). Typically, polarized macrophages could be divided into the classically activated M1 and the alternatively activated M2 phenotypes (17). M1 macrophages are predominantly responsible for antigen presentation, pro-inflammatory, scavenger, and antitumor effects, while M2 macrophages have the biological capacity to inhibit inflammation, promote tissue remodeling, and prevent parasitic infection. In addition, the M2 subtype is implicated in angiogenesis, immune regulation, and tumor progression (18).

Tumor-associated macrophages (TAMs) exhibit significant immunosuppressive effects and belong predominantly to the M2 phenotype of macrophages. M2 executes the pro-tumoral function by promoting tumor growth, immune evasion, angiogenesis, invasion, and metastasis (19, 20). The peroxisome proliferator-activated receptor (PPAR) pathway is a well-known signaling pathway involved in FA metabolism (21). Wu et al. reported that the receptor-interacting protein kinase 3 (RIPK3), a central factor in necroptosis, was downregulated in hepatocellular carcinoma (HCC)-associated macrophages. This increased the accumulation and polarization of M2 TAMs by significantly inhibiting caspase1-mediated cleavage of PPAR and facilitating FA metabolism (e.g., *via* FAO), ultimately leading to accelerated HCC growth (22). Zhang et al. utilized an *in vitro* model to mimic the TAM–HCC interaction in the TME. They found that M2 monocyte-derived macrophages (MDMs) promoted HCC cell migration in an FAO-dependent manner by enhancing interleukin (IL)-1β secretion (23). Sterol regulatory element-binding proteins (SREBPs), a family of membrane-bound transcription factors located in the endoplasmic reticulum (ER), play a central role in regulating lipid metabolism. Liu et al. reported that regulatory T cells (Tregs) suppressed the secretion of interferon (IFN)-γ by CD8^+^ T cells, which would otherwise block the activation of SREBP1-mediated FAS in M2 TAMs. Moreover, SREBP1 inhibition augmented the efficacy of immune checkpoint blockade, suggesting that the combination of targeting Tregs and the lipid metabolism of M2 TAMs could improve the efficacy of cancer immunotherapy (24). Another study revealed that the activity of the mammalian target of rapamycin (mTOR) pathway was elevated in TAMs. Enhanced mTORC1 signaling caused an increase in lipid synthesis within these TAMs (25); the underlying mechanisms, however, require further exploration.

In addition, several studies have shown that the cellular accumulation of lipids was crucial for regulating the function of TAMs. TAMs stimulated and isolated from different mouse tumor models exhibited a reduction in monoacylglycerol lipase (MGLL) expression. Meanwhile, macrophages derived from a transgenic mouse model exhibiting MGLL overexpression were shown to accumulate lipids, and such macrophages preferentially polarized into the M1 phenotype in response to cancer-specific stimuli. Conversely, downregulated MGLL contributed to the functional suppression of CD8^+^ T cells associated with tumor progression. Thereby, the TAM phenotype required a reduction in MGLL expression to stabilize and display immunosuppressive and pro-tumoral functions. Hence, targeting MGLL could be exploited for the treatment of cancer (26). Of note, another study showed that the enrichment of long-chain (LC) omega-3 (ω-3) FAs inhibited the polarization and secretory function of M2 macrophages in a murine prostate tumor model (27, 28). We may therefore hypothesize that the uptake of diverse FA types could give rise to distinct forms of downstream metabolic activity, each exerting different effects on the tumor immune response of macrophages. Moreover, the enzyme-instructed self-assembly of phosphotyrosine-cholesterol was shown to re-educate pro-tumor macrophages into an antitumor phenotype. This phenotype change was achieved by inducing reactive oxygen species (ROS) production and disrupting macrophage-associated filaments, thus inhibiting ovarian cancer progression (29). These findings collectively imply that the underlying mechanism of cholesterol remodeling shapes macrophage effector functions.

### 2.2 T cells in the tumor microenvironment

T cells are predominantly categorized into CD4^+^ and CD8^+^ T-cell subsets; both have crucial functions in the eradication of tumors and pathogens (30). However, the TME has been shown to suppress T-cell function and even remodel their metabolism, resulting in the dysfunction of immune surveillance and the immune evasion of tumor cells (31). CD8^+^ T cells are the most important T-cell subset in the adaptive immune response against tumors. Compared to normal tissues, tumors contain a lower density of CD8^+^ T cells and a reduced CD8^+^ T cell to Treg ratio; these phenomena are associated with poor cancer prognosis (32). CD4^+^ T cells predominantly differentiate into T helper (Th)1 and Th2 cell subtypes. Th1 cells improve the killing activity of CD8^+^ “killer” T cells as well as macrophages, while Th2 cells promote the activation and differentiation of B cells. Thus, the role of CD4^+^ T cells is to influence other immunocytes and their effector functions to prevent infection and tumor growth (11). Aside from the classic T-cell subtypes, other rare T cells also contribute to human immune system homeostasis, including natural killer T cells (NKTs), Tregs, Th9, Th17, Th22, and T follicular helper (Tfh) cells. We will now in turn discuss the lipid metabolism reprogramming capacity of some of these cells within the TME.

#### 2.2.1 CD8^+^ T cells in the tumor microenvironment

CD8^+^ T cells are considered the most important executors of antitumor immunity. Lipids are the essential materials for cell bioactivity, and their depletion in CD8^+^ T cells inhibits cell proliferation and signal transduction. However, this does not mean that excessive lipid production would lead to better cell function. Unraveling the role of lipids in CD8^+^ T cells may be beneficial to furthering our understanding of T-cell function.

Raised FA concentrations in the TME have been shown to activate PPAR-α signaling, which had an essential role in facilitating lipid metabolism and preserving the effector functions of CD8^+^ T cells (33). Nanomedicine, which involves the encapsulation of anti-cancer chemicals and biological tumor necrosis factors (TNFs) in nanoparticles, represents a new form of tumor therapy (34). Kim et al. generated a fenofibrate-encapsulating nanoparticle (F/ANs), the surface of which was modified with an anti-CD3e f(ab′)2 fragment, thus yielding aCD3/F/ANs. PPAR-α and downstream FA metabolism-related genes were overexpressed in aCD3/F/AN-treated T cells. These overexpressed genes resulted in the increased proliferation, killing potency, and infiltration of CD8^+^ T cells in a glucose-deficient environment mimicking the TME (35). This finding, as a new modality of immune metabolic therapy, highlights the potential of nanotechnology to modulate the reprogramming of lipid metabolism and the value of targeting the PPAR pathway in CD8^+^ T cells. In addition, Chowdhury et al. proved that potentiated FAO levels could also enhance the antitumor activity of CD8^+^ T cells in conjunction with anti-programmed cell death protein 1 (PD-1) antibody treatment (36).

However, there is evidence to suggest that lipid accumulation in CD8^+^ T cells contributes to their immune dysfunction. Cluster of differentiation 36 (CD36), also named FA translocase (FAT), is an integral transmembrane glycoprotein expressed in various tissues, which is involved in the high-affinity uptake of long-chain FAs (LCFAs). Increasing evidence has shown that the abnormal expression of CD36 in immunocytes may be involved in tumor-associated processes, such as tumor angiogenesis and tumor immune evasion (33). CD36 mediates the uptake of FAs by CD8^+^ tumor-infiltrating T cells and is also implicated in lipid peroxidation and ferroptosis. In the TME, these phenomena are induced by cholesterol and have been shown to decrease cytotoxic cytokine production by CD8^+^ T cells with their overall antitumor capacity impairment (37). Xu et al. found the emergence of lipoproteins accumulation in CD8^+^ tumor-infiltrating lymphocytes (TILs) within the TME. This was also associated with the increased expression of CD36. In accordance with previous findings, the authors proved that CD36 expression was also correlated with progressive T-cell dysfunction. Mechanistically, CD36 promoted the uptake of oxidized low-density lipoprotein (OxLDL) into T cells and concurrently induced lipid peroxidation and activation of the p38 kinase. Consequently, the inhibition of p38 and resolution of lipid peroxidation restored CD8^+^ TIL functionality. Thus, the OxLDL/CD36/p38 axis promoted intratumoral CD8^+^ T-cell dysfunction in the context of melanoma/colorectal carcinoma (38) and may also serve as a therapeutic target in other tumors.

Senescent T cells exhibit active glucose metabolism but have an imbalance in their lipid metabolism. Tumor cells and Tregs have been shown to drive the expression of group IVA phospholipase A2 (IVA-PLA2). Such increment was responsible for the altered lipid metabolism and the induction of senescence observed in CD8^+^ T cells. Inhibition of IVA-PLA2 reprogrammed the lipid metabolism of effector CD8^+^ T cells, prevented CD8^+^ T cell senescence *in vitro*, and enhanced antitumor immunity and immunotherapy efficacy (in mouse models of melanoma and breast cancer *in vivo*) (39).

In addition, some studies reported that the fluctuation in cholesterol levels had a key role in regulating the immune function of CD8^+^ T cells. Ma *et al.* found that when cholesterol accumulated in the cytoplasm of CD8^+^ T cells, it induced the overexpression of inhibitory checkpoints [PD-1, TIM-3, and lymphocyte activation gene 3 (LAG-3)] in an ER-stress-X-box binding protein 1 (XBP1)-dependent manner, leading to the functional depletion of CD8^+^ T cells. In contrast, lowering cholesterol levels or ER stress could enhance the antitumor function of CD8^+^ T cells (40). Tc9 cells (IL-9-producing CD8^+^ T cells) exhibit stronger antitumor potency compared to conventional CD8^+^ T cells. These cells were shown low levels of PPAR-α/retinoid X receptor (RXR)α, accompanied by a lower expression of the cholesterol synthesis enzymes (HMGCR and SQLE), and a higher expression of the cholesterol efflux enzymes (ABCA and ABCG1). Moreover, the addition of cholesterol-derived oxysterols inhibited IL-9 expression, induced the apoptosis of Tc9 cells, and resulted in their impaired antitumor activity (41). Nevertheless, Yang et al. pointed out that raising cholesterol levels by blocking cholesterol esterification with ACAT1/2 inhibitors facilitated the migration of T-cell receptor (TCR) microclusters to the immunological synapse center (42). The outcome was an increase in the number and activity of anti-melanoma CD8^+^ T cells, as demonstrated both *in vitro* and *in vivo*. These findings highlight the conflicting roles of cholesterol in the regulation of immunocyte function, the intricacies of which require further investigation.

#### 2.2.2 Tregs in the tumor microenvironment

Tregs contribute to immune homeostasis and are essential for orchestrating immune tolerance and the prevention of autoimmune diseases. However, Tregs tend to suppress the antitumor immune response as well as the proliferation of other antitumor immunocytes, such as CD8^+^ T cells and NK cells. Tregs also exert a pro-tumor effect by secreting anti-inflammatory factors like IL-10 and tumor growth factor (TGF)-β, while inhibiting the production of pro-inflammatory cytokines by other immune cells. Thus, Tregs act as negative immune regulators of the antitumor response and promote tumor immune escape and TME maintenance (43).

Xu et al. reported that the Treg-specific ablation of the glutathione peroxidase 4 (GPX4) enzyme repressed melanoma growth and concomitantly promoted antitumor immunity. The mechanism involved in the excessive accumulation of lipid peroxides and ferroptosis in Tregs upon TCR/CD28 co-stimulation (44). FAS mediated by FA synthase (FASN) contributes to the functional maturation of Tregs, with FAO supplying a key energy source for Tregs infiltrating the TME; these features could partly explain why Tregs normally perform their immunosuppressive role in such a glucose-deficient microenvironment (45, 46). CD36 also plays a critical role in Tregs. Wang et al. demonstrated how CD36 depletion decreased the uptake of lipids by Tregs leading to tumor growth deceleration in a melanoma model (33). The authors also observed synergistic effects between the anti-CD36 and anti-PD-1 antitumor therapy. This study indicates that CD36 blockade may represent a powerful enhancer of current immunotherapies by targeting the lipid metabolism of Tregs. Furthermore, another study reported that HCC-associated Tregs expressed PD-1, which promoted the FAO of endogenous lipids by increasing carnitine palmitoyltransferase 1A (CPT1A) protein expression and inducing lipolysis; however, the detailed mechanism has not yet been delineated (47).

Fatty acid-binding proteins (FABPs) are a family of lipid chaperones required for lipid uptake and intracellular lipid trafficking. One of the FABP family members, FABP5, promotes FA absorption from the microenvironment, and FA transportation to specific cellular compartments has been shown to be highly expressed in Tregs. Field et al. showed that in Tregs, the genetic or pharmacologic inhibition of FABP5 function caused mitochondrial changes that were underscored by decreased oxidative phosphorylation (OXPHOS). Consequently, lipid metabolism was impaired, leading to the loss of cristae structure. Interestingly, although FABP5 inhibition in Tregs triggered mitochondrial (mt)DNA release and consequent cGAS-STING-dependent type-I IFN signaling, these changes induced the production of IL-10 by Tregs and promoted their suppressive activity in a murine lymphoma model (48). However, whether long-term mitochondrial alterations mediated by the depletion of FABP5 could lead to increased Treg death remains unknown.

Lim et al. demonstrated that the activity of SREBPs was upregulated in tumor-infiltrating Tregs (49). Inhibiting lipid synthesis and metabolic signaling by targeting SREBPs in the Tregs effectively activated the antitumor immune response without eliciting autoimmune toxicity in the context of melanoma. In addition, SREBP-cleavage-activating protein (SCAP), a downstream target responsible for regulating SREBP activity in Tregs, was identified. The authors demonstrated that the inhibition of the SREBP–SCAP axis attenuated tumor growth and boosted immunotherapy in combination with PD-1 targeting. Moreover, they further showed that Tregs present in the TME exhibited upregulated PD-1 gene expression dependent on SREBP activity and mevalonate metabolism signaling (which led to protein geranylgeranylation), thus identifying a new target for cancer therapy. Similarly, Pacella et al. confirmed that the activation of SREBP promoted lipid synthesis and in turn supported the local proliferation of OX40^+^ Tregs in the TME in a mouse colon carcinoma model and samples from patients with liver cancer (46). OX40, also known as TNF receptor superfamily member 4 (TNFRSF4) and CD134, shapes the lipid composition of Tregs and promotes the proliferation of OX40^+^ Tregs in the TME by inducing FAS and glycolysis.

Regulatory-associated protein of mammalian target of rapamycin (Raptor)/mTORC1 signaling is essential for the suppressive activity of Tregs by promoting cholesterol biosynthesis and lipid metabolism. During the activation of this pathway, mevalonate signaling (which is downstream of the mTOR pathway) is particularly important for the proliferation of Tregs and the upregulation of the suppressive molecules cytotoxic T lymphocyte antigen-4 (CTLA-4) and ICOS to establish Treg functional competency (50). In addition, after selectively deleting ABCG1 in the Tregs of LDL receptor-deficient mice, Cheng et al. observed a 30% increase in Tregs exhibiting intracellular cholesterol accumulation and downregulation of the mTOR pathway, which was responsible for the differentiation of naive CD4^+^ T cells into Tregs. The increased number of Tregs resulted in reduced antitumor T-cell activation and increased IL-10 production, indicating that ABCG1 regulated T-cell differentiation into Tregs (51).

#### 2.2.3 Other T-cell subtypes in the tumor microenvironment

Th2 cells, a subset of CD4^+^ T cells, are significantly involved in the clearance of extracellular parasites as well as asthma and other allergic reactions. Th2 cells have been reported to possess a double-edged effect (52, 53) that depends on the specific kind and stage of the tumor. Moreover, it is conventionally assumed that Th2 cells exhibit tumor-promoting function (53). Of note, PPAR-γ controls the expression of genes associated with lipid metabolism, and it is of tremendous importance for the activation and immune function execution of Th2 cells (52), whereas there is little evidence to prove that this molecule could be influenced by Th2 cells within the TME.

Studies implied the vital role of lipid in the functional maturation of Th17 cells (54). Th17 cells not only are correlated with autoimmune diseases but also play paramount roles against pathogens as well as tumor cells (55). In terms of Th17 cells, they have been proved to fluctuate their number in distinct tumor types. However, its precise mechanism, for instance, how to affect cancers, has not been cleared yet, and it is even contradictory to their immune role referring to a recent concept (55, 56). CD5L (CD5 antigen-like protein) is a member of the scavenger receptor cysteine-rich superfamily involved in lipid metabolism. It exerts the function of inhibiting FASN and is indicated as a regulator for the pathogenicity related to Th17 cells. Such alterations by FASN inhibition may promote RORγt (a transcriptional factor) to bind to the anti-inflammatory genes (*IL10*) whereas preventing it from binding to the *IL17a* and *IL23r* loci (pro-inflammatory genes) in Th17 cells. These changes would eventually cause the pathogenicity of Th17 in the autoimmunity context (57). Nonetheless, the study involved in Th17 lipid metabolic alterations within the TME is in shortage; the exact roles of Th17 in specific tumor types and if the rewiring of lipid metabolism would remodel its immune response are still limited.

Th1 assists cytotoxic cells like NK cells, CTLs, and antigen-presenting cells (APCs) through direct touch or indirect signaling activation. Such effort devotes to elevated immune elimination of pathogens and destructive antitumor immune responses of these antitumor cells (58). Sphingolipids, including two central bioactive lipids, ceramide and sphingosine-1-phosphate (S1P), perform opposing roles in sustaining the survival of tumor cells. Accordingly, some studies implied ceramide acted as an antitumor role, whereas S1P behaved in a pro-tumor manner (59–61). Acid sphingomyelinase (ASM) is a lipid hydrolase enzyme converting sphingophospholipids to ceramides in lysosomes, and Bai et al. reported that elevated ASM bioactivity and ceramide production promoted naive CD4^+^ T cells differentiating into Th1. Moreover, ASM activity also contributed to the expansion of Th17 cells (62).

NKTs, a cluster of CD1d-restricted T cells participating in adaptive and innate immune together, are characterized by recognizing lipid antigens. Lipid metabolism acts as a significant regulator of the cytotoxicity of NKT cells. FAO is of vital importance in maintaining the biofunction of tumor-infiltrating invariant (i)NKTs. Stimulated human (i)NKTs cells utilize fatty acids as the substrates for oxidation more than stimulated conventional T cells, such as CD4^+^ and CD8^+^ T cells. In addition, (i)NKTs display a higher level of FAO and high expression of adenosine monophosphate-activated protein kinase (AMPK) pathway genes (63). Given the complex content in various TMEs, (i)NKTs could also be affected to exert their inherent antitumor function by the blockade of lipid metabolism. However, more evidence would be required to confirm this hypothesis. There is little research focusing on lipid metabolism reprogramming of helper T cells in the TME; perhaps it is noteworthy for us to pay attention to their immune metabolism pattern transition.

### 2.3 Dendritic cells in the tumor microenvironment

DCs are predominantly classified into three categories according to their expression of cell surface molecules and transcription factors: 1) conventional (c)DCs, which can be further subdivided into two subtypes, cDC1 and cDC2; 2) plasmacytoid (p)DCs; and 3) monocyte-derived (mo)DCs. DCs undertake the role of capturing antigens derived from pathogens or tumor cells. They then present the specific antigens to T cells for the activation of the adaptive immune response. Thus, DCs provide the crucial link between innate and adaptive immunity. In addition, DCs release cytokines to help immune effector cells to exert their antitumor effects.

A high lipid content promotes the accumulation of phospholipids and triacylglycerols but not cholesterol and cholesteryl esters in DCs. Intriguingly, these lipid-rich DCs exhibit higher levels of integrins, co-stimulatory molecules, glycoproteins, pro-inflammatory cytokines, and chemokines than DCs with a low lipid content (64, 65).

FAS is crucial for the maturation of DCs as well as their ability to express costimulatory molecules, undergo toll-like receptor (TLR)-mediated activation, and induce T-cell responses. However, the precise roles of FAS and FAO in regulating DC function await to be defined (66, 67). The activation of DCs depends on TLR signaling through which DCs potentiate their glycolysis to produce high levels of pyruvate. As the fuel of mitochondrial respiration, pyruvate is sequentially transformed into acetyl coenzyme A (acetyl-CoA), which is required for FAS and the normal immune function of DCs (66). Li et al. proposed that the uptake of HCC-derived alpha-fetoprotein (AFP) accounted for the reduced expression of CD1 on moDCs (68); this finding was consistent with the results of Santos and colleagues. Santos et al. found that during the early stages of DC maturation, HCC cells could secrete AFP to inhibit FAS and the mitochondrial metabolism of DCs (69). Mechanistically, AFP was shown to downregulate the expression of SREBP-1 and PPAR-γ co-activator-1α (PGC1-α) in DCs *in vitro*. Both SREBP-1 and PGC1-α functioned as regulatory molecules for FAS and mitochondrial metabolism, which were required by DCs for the execution of the antitumor response. These outcomes imply that the curtailed immune function of DCs could be partially attributed to FAS inhibition, thus providing new insights into DC-mediated cancer immunotherapy approaches.

Although the aforementioned research has highlighted the positive effect of high lipid concentrations and altered FA metabolism on DC function, these parameters have also been assigned contradictory roles in the reprogramming of DCs within the TME. These controversial conclusions remind us of the complex signaling networks implicated in the lipid metabolism of DCs. In addition, other potential factors such as the influence of tumor types, DC phenotypes, lipid species, and the interaction between DCs and the soluble components of the TME should also be considered.

Gao et al. reported how blocking FA uptake or impairing lipid synthesis in DCs within a radiation-induced thymic lymphoma model rescued the immunosuppressive state of the TME by improving the T cell-stimulating capacity of DCs (70). Accordingly, Jiang et al. showed that the degree of FASN expression in tumor-infiltrating DCs in ovarian cancer was associated with the advanced clinical phenotype (71). Consecutive activation of FASN in DCs increased lipid assembly, causing abnormal lipid synthesis and lipid accumulation within the cell. This abnormal state may be responsible for the defective ability of DCs to present antigens and activate the antitumor T cell-mediated immune response within the TME. Macrophage scavenger receptor 1 (MSR1) is expressed in DCs and is deemed to be a positive regulator of the immune response by modulating antigen cross-presentation. However, a study has shown that DCs in the TME (of murine colon carcinoma, melanoma, and lymphoma tumor models) increased their expression of MSR1. This gave rise to superfluous lipid uptake and lipid accumulation in these cells (72). The consequences were a reduction in the expression of costimulatory molecules on the DC surface, and in the DC-mediated cytokine production, which ultimately lowered activated T-cell activation. In addition, Yin et al. reported that inactive DCs in the TME were characterized by abnormal lipid accumulation in the cytoplasm (73). They found that tumor cells (murine breast cancer 4T1, cervical carcinoma TC-1, colon carcinoma MC38-OT I, and MC38 and melanoma B16/F10 cell lines) secreted tumor-derived exosomes (TDEs), which arose the PPAR-α-mediated reaction in DCs; in the meantime, PPAR-α also undertook the role of transporting TDEs into DCs. Since PPAR-α signaling acted as the mediator of lipid metabolism, Yin and colleagues in turn demonstrated that PPAR-α contributed to the excessive biogenesis of LDs and concomitantly enhanced FAO in DCs, culminating in DC dysfunction. Conversely, the genetic depletion or pharmacologic inhibition of PPAR-α effectively reversed the TDE-induced immune dysfunction of DC and enhanced the efficacy of immunotherapy. This work uncovered the role of TDE-mediated immune modulation and lipid metabolism in the reprogramming of DCs. Furthermore, it revealed PPAR-α as a stress-induced target, suggesting a novel mechanism by which tumor cells modulated immune cells. Zhao et al. demonstrated that the Wnt5a/β-catenin/PPAR-γ axis was abnormally activated in the context of melanoma (74). This axis was shown to induce FAO in DCs by upregulating the expression of CPT1A, an FA transporter. This in turn increased the levels of the protoporphyrin IX prosthetic group of iIDO (a tryptophan catabolic enzyme) and reduced the expression of IL-6 and IL-12, which normally promote the expansion of Tregs. Furthermore, inhibiting the Wnt5a/β-catenin/PPAR-γ axis not only decreased melanoma progression but also improved the efficacy of anti-PD-1 therapy.

Recently, the identification of certain lipid types (including modified species of lipids) has raised increasing concern due to their ability to cause DC dysfunction. In a study by Ramakrishnan et al., the accumulation of oxidized neutral lipids (e.g., triglycerides, cholesterol esters, and FAs) within DCs, triggered by tumor-derived factors (i.e., originating from the supernatant of EL-4 lymphoma, MC38 colon carcinoma, and CT-26 colon carcinoma cell lineages), was shown to retard the cross-presentation of exogenous antigens. Consequently, the expression of peptide (p)MHC class I complexes on DCs was reduced. Contrary to this phenomenon, the accumulation of non-oxidized lipids did not affect the cross-presentation ability of DCs (75).

There is growing evidence that prostaglandin (PG), the metabolite of arachidonic acid, plays a non-negligible role in the modulation of DC function. Gilardini Montani et al. predicted an increase in the mortality of pancreatic cancer patients following valproic acid (VPA) treatment, due to extensive ER stress and dysregulated choline metabolism in DCs (76). Intriguingly, their investigation of the detailed mechanism found elevated concentrations of prostaglandin E2 (PGE2) (released by VPA-treated cancer cells) in the cellular supernatant. DCs cultured in this supernatant consequently exhibited a lower allostimulatory capacity and an increased ability to release IL-10 and IL-8. These findings suggest that the secretion of PGE2 by the tumor transferred the stress of VPA treatment from the tumor cells to DCs. In agreement with this finding, Amberger et al. designed two new PGE1-containing protocols (Pici-PGE1, Kit M) to generate DC/leukemia-derived DC (DCleu) *in vitro* from leukemic peripheral blood mononuclear cells (PBMCs) or directly from leukemic whole blood (WB) (77). The results showed that PGE1-containing Kit M generated significantly higher amounts of mature DCs from not only leukemic but also healthy PBMCs. Furthermore, it was possible to directly produce DCs from leukemic and healthy WB without triggering their extensive proliferation. Also, compared to the PGE2-containing Kit K, Kit M exhibited higher DC numbers and increased anti-leukemic activity, demonstrating that different subtypes of PG may exert distinct effects on the antitumor process. Additionally, E6, one of the most important oncoproteins associated with human papillomavirus (HPV), was shown to regulate PGE2 synthesis and was associated with the overproduction of PGE2 in HPV-16-positive cervical lesions leading to the inhibition of DC migration (78).

LDs, also named lipid bodies (LBs) are an important cellular organelle involved in the regulation of cellular lipid metabolism by balancing lipid storage and degradation to maintain normal cellular activity. Several lines of evidence suggest that LD metabolic disorders participate in the dysfunction of the DC-mediated immune response. The autocrine secretion of TGF-β2 in the TME by mesothelioma has been shown to account for the abnormal LD accumulation in DCs (79). This suppressed the proliferative and migratory capacities of DCs, preventing their localization to the lymph node to induce CD8^+^ T-cell activation. Tumor-associated DCs (TADCs) with defective antigen cross-presentation ability have been observed in the TME. This defect was partly due to their inability to transport peptide-MHC class I (pMHCI) complexes to the cell surface. Remarkably, DCs in individuals with cancers have been shown to accumulate LBs containing oxidatively truncated (ox-tr) lipids (80). These specific ox-tr-LBs were found covalently bound to the chaperone heat shock protein 70 (Hsp70), thus preventing the translocation of pMHCI to the cell surface. This important research revealed that the species of lipids incorporated into the LBs could determine the role of LBs in the regulation of DC function.

Apart from LDs, lipoprotein metabolism also impacts DC function. Immature moDCs were shown to display notably increased NADPH-oxidase-driven H_2_O_2_-production and LDL uptake (81). These features contributed to the immunosuppressive function of immature moDCs, whereas blocking LDL uptake restored their maturation capacity and attenuated their immunosuppressive properties. Hence, regulating the uptake of LDL may be a potential strategy for modulating the immune function of DCs. However, further research is required to support this hypothesis.

### 2.4 Myeloid-derived suppressor cells in the tumor microenvironment

MDSCs are a differentiated type of myeloid cells that can be divided into three major subpopulations in humans: monocytic (M)-MDSCs (CD14^+^CD15^−^HLA-DR^lo/^ *^−^* cells), polymorphonuclear (PMN)-MDSCs (CD11b^+^CD14*^−^* CD15^+^CD66b^+^ low-density cells), and early-MDSCs (Lin^−^CD11b^+^CD34^+^CD33^+^CD117^+^HLA-DR^lo/^ *^−^* cells) (82). MDSCs have been found in association with various human cancer tissues, where they can act as an independent prognostic factor for the overall survival rate (83). MDSCs usually play an immunosuppressive role in the anti-cancer immune response and support tumor progression and metastasis (84). Therefore, there is an essential need for understanding MDSC function in cancer pathogenesis, with the aim of designing appropriate therapeutic targets or disease markers. Here, we summarize recent research relating to abnormal lipid metabolism in MDSCs.

Cancer-associated MDSCs typically switch their main source of energy from glycolysis to FAO. This metabolic reprogramming is more readily observed in tumor-infiltrating MDSCs, and features increased CD36-mediated FA uptake and higher expression of key enzymes (e.g., CPT1a, medium-chain acyl-CoA dehydrogenase (ACADM), peroxisome proliferator-activated receptor gamma co-activator 1-β (PGC1-β), and 3-hydroxyacyl-CoA dehydrogenase (HADHA)). As a result, the rate of FAO is upregulated, leading to the increased production of immunosuppressive mediators, such as arginase 1 (ARG1) and the cytokines (granulocyte colony-stimulating factor (G-CSF), granulocyte-macrophage colony-stimulating factor (GM-CSF), IL-1β, IL-6, and IL-10) required for the proliferation of MDSCs (85).

In addition to the production of cytokines by MDSCs themselves, the paracrine production of cancer cell-derived G-CSF and GM-CSF also act on the STAT3/5 pathway of tumor-infiltrating MDSCs within the TME. As the downstream target, CD36 was shown to be sequentially upregulated and enhance the uptake of exogenous FAs, thus contributing to the immunosuppressive function of MDSCs (86). Consistently, FATP2 was reported to be exclusively upregulated in the PMN-MDSCs of mice and humans. Meanwhile, the overexpression of FATP2 was proven to be associated with GM-CSF/STAT5 pathway activation. In addition, the absorption of arachidonic acid and the synthesis of its metabolite, PGE2, were implied as being the key players in the FATP2-associated immunosuppressive activity of MDSCs. Conversely, the deletion of FATP2 abolished the suppressive function of MDSCs and even blocked tumor progression in mouse models (EL4 lymphoma, Lewis lung carcinoma, and CT26 colon carcinoma, as well as in a genetically engineered model of pancreatic cancer), when used in combination with immune checkpoint inhibitors (87).

Of note, two soluble mediators, IFN-γ and TNF-α, which are thought to exert pro-inflammatory and antitumor effects, were shown to contribute to the induction of COX2. COX2, as a key enzyme required for PGE2 synthesis, is responsible for the hyperactivation of MDSCs within the TME of patients with ovarian cancer (88). However, this phenomenon was not deemed to implicate either of these factors alone. These findings highlight the overarching role of cytokines in the switching of lipid metabolism, meaning that an approach that targets specific cytokines and their downstream effector molecules could represent a promising therapeutic strategy.

Prior research has implied the potential link between certain cytokines and PGE2 in MDSCs, while other studies underscored the important immune regulatory function of PGE2 *via* another MDSC-relevant axis. The PGE2-forming enzymes, microsomal PGE2 synthase 1 (mPGES1) and COX2, were shown to be highly expressed within Ly-6C^+^ MDSCs in the murine bladder tumor model (89). In contrast, inhibiting the COX2, mPGES1/PGE2 pathway lowered the expression of PD-L1 in these cells and resulted in an elevated number of activated CD8^+^ T cells. The expression of the β2-adrenergic receptor (β2-AR) on MDSCs is positively correlated with breast cancer progression. This phenomenon reveals its role as a pro-tumor factor, which enhances the immunosuppressive activity of MDSCs by reprogramming their metabolism (e.g., by increasing FAO). Interestingly, the increase in CPT1A expression supports the FAO-mediated immunosuppressive effect of MDSCs, consistent with the elevated expression of β2-AR. Moreover, β2-AR signaling is also responsible for increasing the release of the immunosuppressive mediator PGE2 (90). These findings propose an antitumor therapeutic strategy that would rely on lowering the production of PGE2 and its associated upstream and downstream molecules to alleviate the immunosuppressive action of MDSCs.

One study accentuated the difference between the PMN-MDSCs of patients with cancer and healthy individuals by showing that tumor-associated PMN-MDSCs expressed lectin-type oxidized LDL receptor 1 (LOX-1), whereas their normal equivalents did not (91). Accordingly, another finding demonstrated that LOX-1^+^ PMN-MDSCs had a higher level of dichlorodihydrofluorescein diacetate (DCFDA), ARG1, and inducible nitric oxide synthase (iNOS). These LOX-1^+^ PMN-MDSCs possessed the capacity to inhibit the proliferation of CD3^+^ T cells in an ARG1/iNOS-dependent manner. Such suppression of CD3^+^ T-cell expansion in turn signaled a worse prognosis in patients with glioblastoma multiforme (GBM) (92). These results suggest that LOX-1 could also be a prospective antitumor therapeutic target and that the lipoprotein metabolism of MDSCs may contribute to immunosuppression within the TME.

The above studies have shown that lipid metabolism acted as a negative modulator in the TME, leading to the abnormal activation of MDSC. However, PPAR-γ signaling, which also affects the lipid metabolic activity of MDSCs, was demonstrated to support the regular function of lysosomal acid lipase (LAL) in the dampening transendothelial migration (TEM), suppressing the overactivation of the mTOR pathway and preventing ROS overproduction of MDSCs (93). Since these MDSC-associated properties could promote tumor progression, this study provides a mechanistic basis for targeting MDSCs to reduce the risk of cancer initiation, growth, and metastasis.

### 2.5 Natural killer cells in the tumor microenvironment

NK cells, working as an indispensable component of the human innate immune, possess direct killing property and participate in anti-tumor and anti-virus infection. NK cells exert their immune function by releasing cytotoxic granules, mediating the antibody-dependent cell-mediated cytotoxicity (ADCC) effect, and priming death receptor signal of targeted cells to sustain immune homeostasis (94). The research about NK cell lipid metabolism alteration has been referred to below.

In the setting of the postoperative period after colorectal cancer resection, granulocytic MDSCs expanding after surgery induced the expression of scavenger receptors (SR) such as MSR1, CD36, and CD68 in NK cells. The changes resulted in intracellular lipid accumulation with diminished NK cell ability to release granzyme B and perforin, eventually contributing to NK cell dysfunction and colorectal cancer relapse (95). Regarding the finding of Bonavita et al., tumor-derived PGE2 selectively acted on EP2 and EP4 expressed on NK cells to reshape immunosuppressive TME and consequently promoted tumor (colorectal carcinoma and melanoma) immune evasion (96).

## 3 Hypoxia in the tumor microenvironment induces lipid metabolism reprogramming in immunocytes

**Figure 2:**
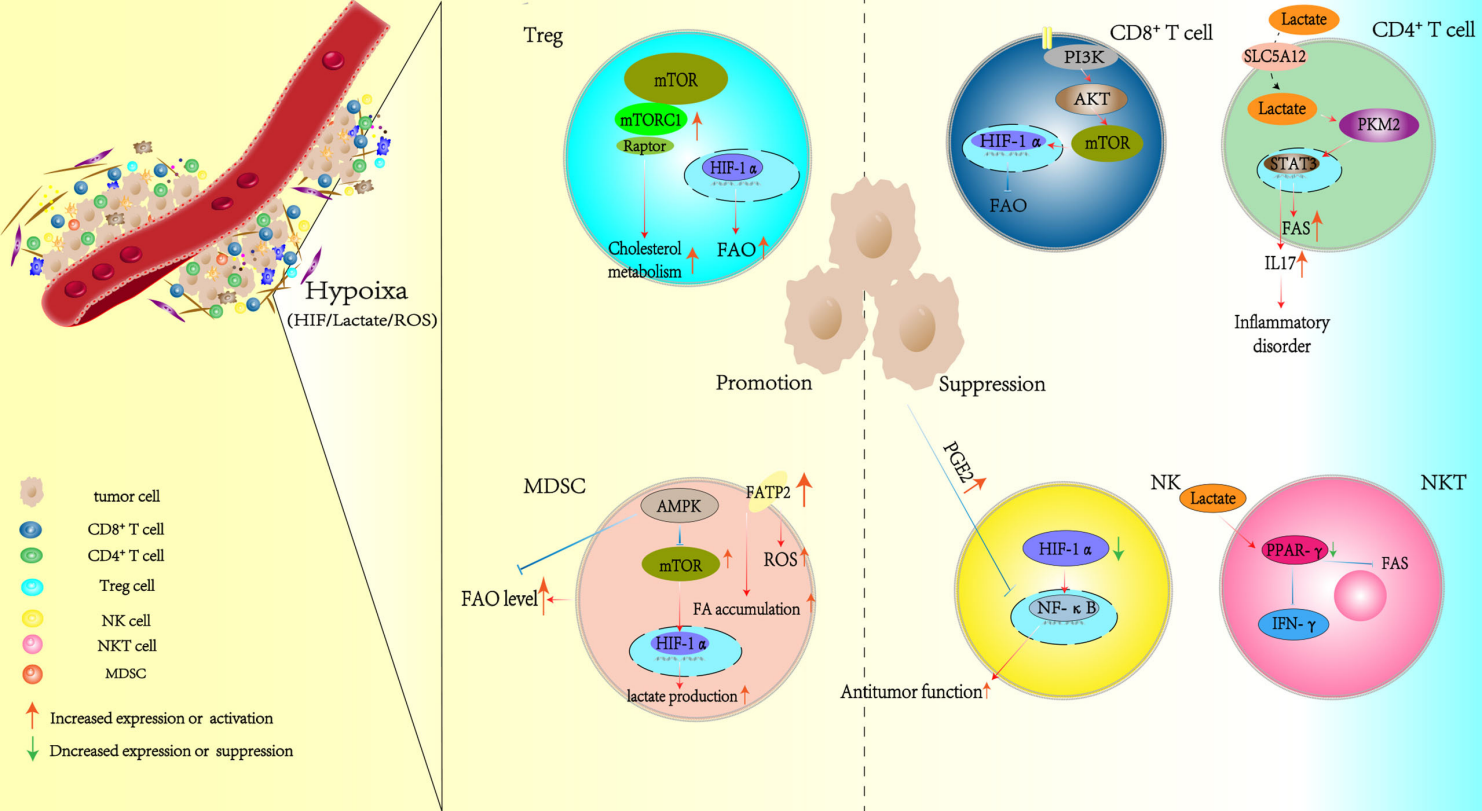
Hypoxia alters the lipid metabolism patterns of immunocytes in the TME. TME, tumor microenvironment.

Under normal physiological conditions, oxygen is taken up by mitochondria to participate in OXPHOS, which provides ATP for typical cellular functions. The extensive depredation of tumor cells and their unlimited growth cause the release of non-cellular components into the TME. These include cytotoxic metabolites. The metabolites ROS (97), lactic acid, and tumor-derived negative regulatory molecules collectively impair the effector function of immunocytes. Although cells possess the flexibility to adapt to an altered milieu, they inevitably sacrifice some bioactivities to guarantee survival. Hypoxia is one of the stressors within the TME that has a significant effect on tumor outcome (98). Hence, broadening our understanding of how hypoxia affects the function of immunocytes is necessary. Here, we have assembled findings related to the switch in lipid metabolism that occurs in immune cells as a result of hypoxia. In addition, we also discuss the molecular targets and pathways that could be utilized to alleviate immune cell dysfunction, which is a result of the hypoxia-driven reprogramming of lipid metabolism (**Figure 2**).

Hypoxia represents an obstacle for the immune system in the fight against tumors (99). Among the molecules that contribute to the hypoxia-mediated state of immune inhibition, hypoxia-inducible factor (HIF), ROS (elevated production in anoxic conditions), and lactic acid (lactate) (which mostly contributes to an acidic environment) are predominantly responsible for the induction of immune cell dysfunction (100).

The hypoxic TME has been shown to reduce the expansion of CD8^+^ T cells, in addition to impairing the maturation and activity of DCs and NK cells; in contrast, Tregs and MDSCs remain highly active in the same context (101, 102). HIF is a transcription factor activated by a low-oxygen microenvironment and is composed of two subunits: HIF-α (which occurs in the form of three isoforms: HIF-1α, HIF-2α, and HIF-3α) and HIF-β (103). HIF is reported to promote lipid peroxidation and ER stress and recruit Tregs, M2 macrophages, and MDSCs to construct an immunosuppressive TME (6). However, a recent finding by Velica *et al.* proposed that it was HIF-2α, not HIF-1α, that triggered the increased cytotoxic differentiation and cytolytic function of CD8^+^ T cells against tumor targets (104). This suggests that distinct subtypes of HIF perform divergent roles in response to immunocyte stressors.

The differentiation of naïve T cells into effector T cells (Teffs) by TCR/CD28 co-stimulation requires PI3K/Akt/mTOR signaling. HIF-1α locates downstream of this pathway, and once HIF-1α is activated, it consequentially triggers aerobic glycolysis and amino acid metabolism in Teffs while suppressing FAO (105). As a result, HIF-1α is often upregulated under such circumstances, and Teffs ultimately exhibit immune dysfunction. These phenomena may suggest that strong FAO suppression makes a potential contribution to Teff dysfunction in the TME.

It appears that the adaptation of Tregs to the TME depends not only on their glycolysis but also on FAO (98). mTORC1 signaling impairment was shown to compromise *de novo* lipid synthesis in CD3^+^ T cells. Such altered lipid metabolism was associated with the PI3K/mTOR/HIF-α axis, which in turn affected CD3^+^ T-cell adaptation to hypoxic conditions and their execution of immune regulation *in situ* (106). Earlier, we also reviewed that Raptor/mTORC1 signaling in Tregs could promote cholesterol biosynthesis and support the immunosuppressive function and the activation of Tregs. These observations indicate that mTOR signaling plays a previously underestimated role in T cells (and possibly in other immune cells) by bridging HIF and lipid metabolism. In addition, Miska et al. unveiled that HIF-1α-directed glucose away from mitochondria; thus, Tregs consequently depended on FAs for their energy in the hypoxic tumor tissue. Conversely, inhibiting lipid oxidation enhanced the overall survival of glioma-bearing mice; however, the direct link between HIF-1α and lipid oxidation remained unclear (107). PGE2 is upregulated in the TME (thyroid cancer background), and a high concentration of PGE2 was shown to result in the NF-κB pathway-mediated suppression of NK cells (108). Another study by Ni et al. clarified that the inhibition of HIF-1α enhanced the IL-18-driven activation of the NF-κB pathway. This activated axis in turn elevated the expression of activation markers and effector molecules in tumor-infiltrating NK cells, thus promoting tumor suppression (109). These results reveal the potential link between PGE2, HIF-1α, and NF-κB signaling in NK cells; however, further supporting research is required.

There are dramatic differences in the metabolism of M1 and M2 macrophages. M1 macrophages mainly derive their energy from glycolysis and the existing blockade of the tricarboxylic acid (TCA) cycle, which results in the intracellular accumulation of itaconate and succinate. Succinate overproduction leads to HIF-1α stabilization and, in turn, activates the transcription of glycolytic genes. Such genes are responsible for maintaining the glycolytic metabolism of M1 macrophages. On the contrary, M2 cells are more dependent on OXPHOS for their energy. Moreover, their TCA cycle is intact and provides the substrates with complexes of the electron transport chain (110). The effect of hypoxia on macrophages is also vague. A study has reported the positive effect of hypoxia on macrophages, which sustained their inflammatory responses and migratory capacity (111). In contrast to this report, hypoxia has also been shown to impair macrophage pro-inflammatory activity by eliciting energy metabolism disorder (EMD), which particularly affected glucose metabolism (112, 113). These results have predominantly occurred in the M1 subtype. The possible explanation for these disparate conclusions may be related to the duration of low-oxygen conditions and the preferable differentiation of macrophages from M1 to M2 within the TME (114). The effect of hypoxia on M2 macrophages is well studied and is deemed as a driver of M2-mediated pro-tumor activity (101). Regrettably, although numerous studies have established the mechanism for how hypoxia affects the glucose metabolism of macrophages in the TME, which ultimately results in macrophage dysfunction, the investigations of lipid metabolism alteration remain limited.

Hypoxia was proven to aggravate tumor progression with aberrant PPAR signaling activation. In the setting of glioma, PPAR signaling hyperactivation was shown to immunosuppress by promoting Treg expansion (115). Since PPAR largely regulates cellular lipid metabolism, aiming at an undetected target stressed by hypoxia through this signaling to mediate Treg lipid metabolism and in turn support the immunosuppression function of Tregs may be a prospective research orientation. Liu et al.> proposed the excessive production of IDO1 in DCs within a hypoxic milieu (116). This finding appears to be similar to the result we previously cited (74) and may be the clue to explaining the underlying correlation between hypoxia and the Wnt5a/β-catenin/PPAR-γ signaling pathway, which may mediate the reprogramming of lipid metabolism in DCs within the hypoxic TME.

Lactate is the main metabolite of glycolysis. Hypoxia has been shown to greatly increase lactate levels, causing the acidification of the TME. Such an acidic milieu consequently facilitates tumor progression and suppresses antitumor immunity through T-cell inhibition and PD-L1 upregulation (117). SLC5A12, a lactate transporter, mediates lactate uptake by CD4^+^ T cells and induces the reshaping of their effector phenotype resulting in increased IL-17 production *via* the nuclear PKM2/STAT3 pathway. Of note, SLC5A12-mediated lactate uptake into CD4^+^ T cells also promotes FAS. Such abnormal lactate content eventually leads to the onset of chronic inflammatory disorders by CD4^+^ T cells (118); however, its relationship with the TME demands more research. mTOR signaling is inhibited by AMPK activation and regarded as an effective activator of HIF-1α signaling as well as lactate production. In addition, AMPK was also shown to restrict FAO. Interestingly, the extent of both lactate production and FAO levels is increased in MDSCs within the TME, which is beneficial for their activity. These findings suggest that activators of AMPK signaling could represent promising MDSC-targeting therapeutic candidates (119). Moreover, the acidic milieu of mesothelioma was shown to trigger DC dysfunction and alter the T cell-mediated immune response through a TGF-β2-dependent mechanism (79). In this context, DC anergy was tightly associated with intracellular LD accumulation and the metabolic rewiring of DCs. Moreover, the aggregation of lactic acid in the TME reduced PPAR-γ expression levels, thus diminishing lipid synthesis and IFN-γ production in invariant (i)NKT cells (120). Conversely, utilizing a PPAR-γ agonist successfully reversed these phenomena and strengthened the antitumor activity of (i)NKT cells in the context of melanoma. Since there are few studies about lactate-induced abnormal lipid metabolism in different immunocyte types within the TME, more effort would be required to progress this field of research.

ROS is easily produced under hypoxic anoxic conditions. A report by Adeshakin et al. proposed the link between ROS and lipid metabolism (121). FATP2 expression in the MDSCs was shown to increase when these cells were present within the TME (thyroid cancer). Meanwhile, the blockade of FATP2 expression in MDSCs by lipofermata lowered their intracellular lipid content, reduced ROS concentration, blocked their immunosuppressive function, and consequently inhibited tumor growth.

## 4 Current immunotherapies and reagents targeting the lipid metabolism of immunocytes

In recent years, immunotherapy has shown an excellent performance in the treatment of various types of cancer. Generally speaking, current efforts in cancer immunotherapy fall into three main approaches (122): 1) the blockade of immune checkpoints such as anti-PD-1/PD-L1 and anti-CTLA-4 to restore or potentiate the antitumor effect of immune cells (123); 2) adoptive cellular therapy, including the use of TIL therapy (124), engineered T-cell therapy (e.g., chimeric antigen receptor [CAR] T-cell therapy and TCR therapy (125)), CAR-NK cell therapy (126), and CAR-macrophage therapy (127); and 3) therapeutic cancer vaccines (128). Although immunotherapy represents a remarkable breakthrough in the treatment of cancer, it still has limited success in certain types of tumors. Tumor resistance, non-responsiveness, and recurrence following immunotherapy could in part explain why existing immunotherapeutic methods are not as effective against cancers as anticipated. Hence, novel therapeutic targets are urgently needed to complement existing forms of immunotherapy. Since the study of immune cell metabolism has received increasing attention, certain biomarkers have also been established as new checkpoints associated with metabolic activity in specific immunocytes. Targeting these markers either alone or in combination with other forms of immunotherapy could contribute to the dysregulated immune situation reversing. Here, we describe the molecular targets that participate in immunocyte lipid metabolism and introduce the drugs that could be used to modulate these targets in favor of the antitumor immune response (**Table 1**).

**Table 1:** Current tumor-suppressor reagents that regulate immune cell function by modulating their lipid metabolism.

Reagent	Target molecule	Effect on lipid metabolism in immunocytes	Influence on immunocytes in the TME	Reference
Etomoxir	CPT1A	Inhibition of FAO	Inhibited the infiltration of MDSCs; potential of suppressing pro-tumor capacity of TAMs	(85, 129)
Nivolumab; pembrolizumab; atezolizumab 2	PD-1/PD-L1	Inhibition of FAO; promotion of cholesterol content	Enhanced immune response of immunocytes such as CD8^+^ T cells	(47, 130)
Ipilimumab	CTLA-4	Interfered with T-cell FAO process	Enhanced antitumor response of T cells	(131)
_	CD36	Inhibition of FA uptake and accumulation	Enhanced intra-tumoral CD8^+^ T-cell effector function; ablated the function of intra-tumoral Tregs	(33, 37)
C75	FASN	Inhibition of fatty acids synthesis	Reduction of IL-1β, TNF-α, IL-6, and IL-10 levels in macrophages	(132)
AICAR	_	Elevation of fatty acid oxidation of CD4^+^ T cells	Specifically enhanced the expansion of Treg cells; impairment of Th17 generation	(133)
RGX-104	LXR	Driving *Apoe* and genes involved in cholesterol, fatty acidic transcriptional activation	Activation of LXR/ApoE axis elicited robust anti-tumor responses; enhanced T-cell activation; suppressed survival and immunosuppressive function of MDSCs	(134)
Atorvastatin	mTOR	Restrained cholesterol content	Downregulated the expression of co-inhibitory receptors in T cells with constant secretion of IL-2	(135)
NS-398	COX-2	Inhibition of PGE2 production	Enhanced the antitumor potency of DCs	(136)
Saponin-based adjuvants	_	Induction of intracellular LBs	Elevated cross-presentation and T-cell activation function of CD11b^+^ DCs	(137)

TME, tumor microenvironment; FAO, fatty acid oxidation; MDSCs, myeloid-derived suppressor cells; TAMs, tumor-associated macrophages; FA, fatty acid; DCs, dendritic cells; LBs, lipid bodies.

In lung carcinoma and colon adenocarcinoma, etomoxir was shown to target CPT1A to inhibit FAO of MDSCs and reversed its tumor-promoting effects by abrogating the infiltration of MDSCs into the tumor (85). In addition, Su et al. demonstrated that high CTP1A expression modulated TAMs to form a pro-tumor subset (129). PD-1/PD-L1 is ubiquitously expressed on immunocytes. Although the detailed mechanism regarding how PD-1/PD-L1 affects immunocyte immune function has been well studied, new research has uncovered that the drugs targeting PD-1/PD-L1 (e.g., nivolumab, pembrolizumab, and atezolizumab) simultaneously dampen their FAO levels; this effect partially enhanced the response of immune cells (e.g., CD8^+^ T cells) that were generally inhibited in the TME (47, 130). Of note, in the setting of HCC, the expression levels of PD-1 in HCC patients were reported to be markedly higher than in healthy donors. This phenomenon implies that targeting PD-1 to alleviate the suppression of antitumor immune function in immunocytes (within the hepatic TME) could potentially be achieved by regulating their lipid metabolic patterns (138). Moreover, ipilimumab targets CTLA-4 to positively regulate the antitumor immune response and was also reported to interfere with the FAO process in T cells (131). The CD36 inhibitor and the anti-CD36 antibody are proposed as novel therapeutic molecules used to enhance intratumoral CD8^+^ T-cell effector function (37) and ablate the immunosuppressive function of intratumoral Tregs (33); related research, however, is still in the preclinical stage. In addition, C75, as a FASN inhibitor, was shown to reduce IL-1β, TNF-α, IL-6, and IL--10 levels in macrophages; further results are anticipated (132).

Gualdoni et al. found that AICAR, an AMP analog, modulated the ratio of Tregs/Th17 cells by regulating FAO in CD4^+^ T cells (133). Mechanistically, AICAR directly activated AMPK and specifically induced Treg expansion while impairing the generation of Th17 cells. This phenomenon was associated with an increase in FAO in CD4^+^ T cells. LXR (liver-X nuclear receptor) is a member of the nuclear hormone receptor family. As a transcription factor, LXR has the capacity to drive the transcriptional activation of *APOE* and other genes involved in cholesterol, FA, and glucose metabolism to limit MDSC expansion. Additionally, the activation of the LXR/APOE axis was shown to elicit robust antitumor responses in various tumor models and human tumor cell lineages. RGX-104, an agonist of LXR, was proven to suppress the survival and immunosuppressive function of MDSCs; this research was in the Phase I a/b stage (134).

Atorvastatin, one of the classic cholesterol-lowering drugs, was shown to downregulate the expression of co-inhibitory receptors in T cells accompanied by the prolonged secretion of IL-2, as observed in chronic infections such as HIV, hepatitis C virus (HCV), and cancer. However, atorvastatin treatment actually compromised T-cell proliferation and reduced their capacity to secrete TNF-α; related mechanisms were also involved in the inhibition of Ras-activated MAPK, PI3K-Akt, and subsequent mTOR signaling pathways (135).

The regulation of arachidonic acid metabolism in the TME is also thought to have a positive effect on tumor eradication. Pandey et al. found that the augmented secretion of PGE2 by tumor cells inhibited DC function (136). They utilized the COX-2 inhibitor, NS-398, to reduce PG synthesis and consequently elevated the antitumor potency of DCs by enhancing their immune activity. The mechanism may involve an increased content of the classical DC-lineage-specific transcription factor Zbtb46, as well as a decrease in extracellular signal-regulated kinase (ERK)/the cyclic AMP response-element binding protein (CREB) signaling, which promoted IL-10 synthesis.

Of note, a study showing that the elevated intracellular LB content of CD11b^+^ DCs, which was induced by saponin-based adjuvants (used for animal and human cancer vaccines), could improve DC-mediated cross-presentation and T-cell activation *in vitro* and *in vivo* (137).

## 5 Discussion

Since the notion of the TME was proposed, multiple studies have uncovered the role of the TME in tumor initiation, progression, metastasis, and its response to treatment. TME components include tumor-infiltrating immune cells (recruited in response to tumor antigens), chemokines, and other soluble factors, all of which are strongly associated with cancer prognosis. In recent years, immunometabolism has gained increasing attraction for its implication in the regulation of immune cell function (139). Furthermore, evidence of immunometabolism reprogramming has been uncovered in various types of tumors. The studies we have listed in this review collectively reveal the true value of targeting the TME-induced lipid metabolism reprogramming of immunocytes. Hence, in this section, we summarize the lipid metabolism-associated challenges awaiting to be addressed in each type of immunocytes within the TME by future research.

TAMs tend to differentiate into a pro-tumorigenic, immunosuppressive phenotype, which is associated with an M2 signature within the TME. Because of the TME stressor, the altered lipid metabolism of TAMs preferentially favors macrophage polarization into M2 rather than the M1 subset, sequentially contributing to tumor evasion and progression. Nevertheless, some TME-residing macrophages retain their antitumor functions in the TME. Thus, promoting the M1 macrophage immune response while suppressing the activity or expansion of the M2 macrophages could represent a plausible therapeutic strategy.

In humans, CD8^+^ T cells perform the primary antitumor role, and their function is universally suppressed within the TME. To date, a consensus on how the reprogramming of lipid metabolism impacts CD8^+^ T-cell functions has not been reached. As for Tregs, prior research has demonstrated that the lipid metabolism reprogramming of these cells could also be targeted to improve the immunogenicity of tumors. However, the exact mechanism behind how altered lipid metabolism modulates Treg function within the TME of each tumor type remains uncertain. Moreover, the prevalence of altered lipid metabolism in rare T-cell subsets is also unclear. Thereby, more research is needed to clarify the processes involved in the lipid metabolic rewiring of T cells within the TME.

Research on DC dysfunction associated with the immune evasion of tumor cells has raised concerns, especially as the mechanisms implicated remain elusive. DC metabolism controls the development, polarization, and maturation processes within these cells and provides energy to maintain their function. However, the immune activity of DCs is generally inhibited in the TME, while many relevant metabolic pathways are also strongly altered. Thus, it is rational for us to assume the existence of the unknown mutual interaction between the inhibition of immune function and lipid metabolism alteration. Hence, the discovery of novel therapeutic targets associated with lipid metabolic changes in DCs would be of great value. Encouragingly, prior research has already demonstrated the potential benefits of targeting the metabolic switch of tumor-associated DCs. Based on the research progress that we have summarized, we envision that the development of tailored DC-associated therapies for the treatment of patients with specific tumors could one day become a reality.

MDSCs represent one of the major immunosuppressive immunocytes that reside within the TME (140). The quest to eliminate the immunosuppressive function of MDSCs is a promising field of tumor therapy. In-depth research of MDSCs has revealed prospective insights into the metabolic alterations (including changes in lipid metabolism) affecting these cells; these findings could therefore unveil novel ways of eliminating the immunosuppressive function of MDSCs. Studies have shown that the direct inhibition of MDSCs interacted with immune checkpoints on antitumor immunocytes or immediately restricted the secretion of immunosuppressive mediators (such as IL-10, TGF-β, and PGE2), which may be a thinkable method to mitigate such immunosuppressive milieu contributed by MDSCs. However, research is largely limited to a small subset of specific tumors; thus, more attention needs to be devoted to this area of study.

Regrettably, at present, there is insufficient research on the lipid metabolism reprogramming of NK cells within the TME. Adoptive NK cell transfer therapy, one of the most used NK cell-related immunotherapies, still suffers from limitations such as the challenge of producing sufficient NK cell numbers to efficiently recognize and target the tumor or the slow migration of adoptively transferred NK cells toward the tumor site. Thus, investing in the study of the lipid metabolic reprogramming of NK cells in the TME may improve therapeutic NK regimens in cancer.

The TCA cycle is the major method for ATP production in eukaryotes. A low-oxygen milieu orchestrated by the TME would inevitably affect glucose metabolism and, in turn, FAO and FAS. Moreover, the drop in ATP levels would shift metabolism in favor of the consumption of cholesterol and other lipid species. Since lipids maintain multifarious activities that are necessary for cell survival, the alteration of lipid metabolism in immunocytes would inevitably lead to immune dysfunction and tumor evasion in the TME. Of note, the unknown effect of the shift in lipid metabolism in a low-oxygen environment may be easily masked and underestimated, due to our major focus on glucose metabolism. Hence, the investigation of how hypoxia impacts the lipid metabolism of immunocytes within the TME is worth considering for the development of novel antitumor therapies.

Immunotherapy represents a major breakthrough in the improvement of the overall survival (OS)/relapse-free survival (RFS) of patients with cancers in recent years. However, its limitations, arising from tumor heterogeneity, drug resistance, and non-responsiveness, in addition to the risk of autoimmune disease induction, still need to be overcome. Encouragingly, from the research summarized in this review, we would hypothesize that immunotherapy and drugs regulating immune cells’ lipid metabolism could represent complementary approaches to rescue the antitumor function of antitumor immunocytes or inhibit the immunosuppressive function of pro-tumor immune cells in the TME. However, there are still some challenges for relevant research at the present stage. Firstly, prior research only focused on one specific tumor type; thus, the universality of such drugs needs more exploration since distinct tumors vary in their intra-tumoral compositions. In addition, few studies examined abnormal lipid metabolism within immunocytes in various TMEs from a comprehensive perspective. Thus, considerable effort will be required to establish solid evidence in this field and apply this therapeutic method to distinct tumor categories. Secondly, current cancer treatment regimens only focus on one type of immunocyte. Hence, it is often not possible to determine whether the function of non-targeted immune cells would be affected when employing immunomodulating drugs. These problems need to be taken into consideration to minimize the unknown side effects of novel drugs and optimize therapeutic strategies for cancer patients.

Collectively, our review unveils a largely unexplored and tangible approach to the treatment of patients with cancer. By concentrating on the studies of lipid metabolism reprogramming in immunocytes within the TME, we unveil part of the mechanisms that contribute to this metabolic alteration. Ultimately, we hope that this review will prove beneficial for the development of novel effective antitumor therapies in the near future.

## Author contributions

MZ and TW wrote the draft manuscript. XZ and DG made critical revisions to the manuscript. All authors contributed to the article and approved the submitted version.

## Funding

This study was supported by grants from the National Natural Science Foundation of China (No. 81901571 to DG) and the Henan Provincial Science and Technology Research Project (No. 222102310564 to XZ, No. SBGJ202103056 to DG).

## Conflict of interest

The authors declare that the research was conducted in the absence of any commercial or financial relationships that could be construed as a potential conflict of interest.

## Publisher’s note

All claims expressed in this article are solely those of the authors and do not necessarily represent those of their affiliated organizations, or those of the publisher, the editors and the reviewers. Any product that may be evaluated in this article, or claim that may be made by its manufacturer, is not guaranteed or endorsed by the publisher.

## Glossary

**Table:**

TME	tumor microenvironment
OXPHOS	oxidative phosphorylation
FAO	fatty acid oxidation
FAS	fatty acid synthesis
LD	lipid droplet
DC	dendritic cell
NK	natural killer cell
MDSC	myeloid-derived suppressor cell
N	neutrophils
TAM	tumor-associated macrophage
PPAR	peroxisome proliferator-activated receptor
RIPK3	receptor-interacting protein kinase 3
HCC	hepatocellular carcinoma
MDM	M2 monocyte-derived macrophage
MGLL	monoacylglycerol lipase
FAs	fatty acids
SREBP	sterol regulatory element-binding protein
IFN-γ	interferon-γ
CTL	cytotoxic T lymphocyte
NKT	natural killer T cell
T_reg_	regulatory T cell
Th	helper T cell
Tfh	follicular helper T cell
OxLDL	oxidized low-density lipoprotein
TGF-β	transforming growth factor-β
FASN	fatty acid synthase
CPT1A	carnitine palmitoyltransferase-1A
FABP	fatty acid-binding proteins
AMPK	adenosine monophosphate-activated protein kinase
AFP	alpha-fetoprotein
LB	lipid body
NOS	nitric oxide synthase
HIF	hypoxia-inducible factor
Teff	effector T cells
